# Design and Optimization of W-Mo-V High-Speed Steel Roll Material and Its Heat-Treatment-Process Parameters Based on Numerical Simulation

**DOI:** 10.3390/ma18010034

**Published:** 2024-12-25

**Authors:** Zhiting Zhu, Mingyu Duan, Hao Pi, Zhuo Li, Jibing Chen, Yiping Wu

**Affiliations:** 1 School of Mechanical Engineering, Wuhan Polytechnic University, Wuhan 430023, China; 2 School of Material Science and Engineering, Huazhong University of Science & Technology, Wuhan 430074, China

**Keywords:** W-Mo-V HSS, JMatPro software, austenitic, martensitic transformation temperature, heat treatment

## Abstract

W-Mo-V high-speed steel (HSS) is a high-alloy high-carbon steel with a high content of carbon, tungsten, chromium, molybdenum, and vanadium components. This type of high-speed steel has excellent red hardness, wear resistance, and corrosion resistance. In this study, the alloying element ratios were adjusted based on commercial HSS powders. The resulting chemical composition (wt.%) is C 1.9%, W 5.5%, Mo 5.0%, V 5.5%, Cr 4.5%, Si 0.7%, Mn 0.55%, Nb 0.5%, B 0.2%, N 0.06%, and the rest is Fe. This design is distinguished by the inclusion of a high content of molybdenum, vanadium, and trace boron in high-speed steel. When compared to traditional tungsten-based high-speed steel rolls, the addition of these three types of elements effectively improves the wear resistance and red hardness of high-speed steel, thereby increasing the service life of high-speed steel mill-roll covers. JMatPro (version 7.0) simulation software was used to create the composition of W-Mo-V HSS. The phase composition diagrams at various temperatures were examined, as well as the contents of distinct phases within the organization at various temperatures. The influence of austenite content on the martensitic transformation temperature at different temperatures was estimated. The heat treatment parameters for W-Mo-V HSS were optimized. By studying the phase equilibrium of W-Mo-V high-speed steel at different temperatures and drawing CCT diagrams, the starting temperature for the transformation of pearlite to austenite (A_c1_ = 796.91 °C) and the ending temperature for the complete dissolution of secondary carbides into austenite (A_ccm_ = 819.49 °C) during heating was determined. The changes in carbide content and grain size of W-Mo-V high-speed steel at different tempering temperatures were calculated using JMatPro software. Combined with analysis of A_c1_ and A_ccm_ temperature points, it was found that the optimal annealing temperatures were 817–827 °C, quenching temperatures were 1150–1160 °C, and tempering temperatures were 550–610 °C. The scanning electron microscopy (SEM) examination of the samples obtained with the aforementioned heat treatment parameters revealed that the martensitic substrate and vanadium carbide grains were finely and evenly scattered, consistent with the simulation results. This suggests that the simulation is a useful reference for guiding actual production.

## 1. Introduction

With the modernization of the world, industrial technology automation, high-strength, high-surface-quality steel-plate steel bar production continues to increase, the plant working conditions are an increasingly complex and harsh environment, and the production of steel directly in contact with the quality of the work rolls directly affect the quality of the steel and mill life. It has been very difficult for the traditional high-chromium cast iron rolls and nickel-containing cast iron rolls to meet the production requirements, and therefore, the study of the improvement in the material properties of the rolls in order to meet the actual industrial production demand, is difficult [1,2,3]. So, mainly used in the preparation of high-alloy tool steel for cutting metal tools, high-speed steel is used for roll materials [4]. Compared with traditional roll materials, HSS has excellent hardness, strength, wear resistance, red hardness, and fracture toughness [4,5,6]. In recent years, with the development of rolling technology and the increasing demand for high-quality steel in the world, improving the surface quality and service life of mill rolls has been an important hot topic in the rolling industry. Therefore, the properties such as hardness, wear resistance, and surface roughness of HSS rolls are improved by adjusting the strong carbonization-forming elements such as W, Mo, V, and Cr in HSS rolls and optimizing the roll preparation process and heat treatment process [1,7,8,9].

Li et al. [10,11] used laser additive manufacturing to prepare Nb element addition at 0~2 wt.% of W-Mo-V; HSS can effectively improve its wear resistance and microhardness, and the resulting HSS organization is also more fine and uniform, and at the same time, control of the Nb/C ratio can effectively ensure the secondary hardening ability of HSS rolls. The addition of Nb can also effectively improve the uniformity of the oxide film formed on the wear surface, and the surface roughness and other properties. Xu et al. [12] found that the contained V element gives higher high-speed steel, due to the high hardness of vanadium carbide and good morphology and other characteristics, so it has excellent wear resistance, and can effectively resist the abrasive particles of the micro-cutting. Zhang et al. [13] studied W, Co, and Ni on the Fe-C-V-Cr-Mo-based high-speed steel carbide and alloy hardness effect. The results of the study show that the W element is favorable for the formation of MC carbides, thus effectively improving the hardness of high-speed steel. In addition, Xiao et al. [14] mixed commercial high-speed steel powder with W-Mo-V high-speed steel powder to produce a mixed material, and found that its hardness and wear resistance were both improved. This study also used commercial high-speed steel powder as raw material to formulate W-Mo-V high-speed steel with appropriate alloy ratios. Efremenko et al. [15] showed the interaction between carbon and boron in alloy steels, so that the addition of the element boron affects the microstructure of the steel, to some extent. At the same time, different studies have shown, respectively, that niobium or trace boron elements are added to HSS to improve the mechanical properties of HSS [11,16], and this paper is designed to add these two trace elements and, at the same time, to find their suitable ratios to improve the quality of HSS rolls. In high-speed steel rolls in actual industrial use, the wear of its surface is also related to the carbides formed by its various alloying elements [17], so adjusting the proportion of alloying elements contained in high-speed steel plays an important role in improving the mechanical properties of high-speed steel rolls.

Furthermore, in addition to the influence of alloying elements, high-speed steel roll preparation and heat treatment are critical for improving roll performance. The centrifugal casting method is widely used in the preparation of HSS rolls, due to its high productivity and cost advantages; in addition to electro slag remelting, the continuous pouring process (CPC) of cladding, hot isostatic pressing and injection molding are also used in the preparation of HSS rolls [9]. After selecting a reasonable preparation method, the appropriate heat treatment process to improve the performance of high-speed steel rolls plays an extremely important role. Peng et al. [18] found that, through heat treatment to strengthen the matrix without reducing its toughness, one method is to refine the austenite grain, and then, through the tempering of the coarse residual austenite into martensite, to select the appropriate three tempering temperatures and time. After three times, the compressive strength can be obtained. Tempering, an excellent combination of compressive strength, strain at break, flexural strength, and Rockwell hardness of 3245 MPa, 30.6%, 2094 MPa, and HRC 62.2, respectively, was obtained. Pan et al. [19] found that HSS prepared by jet molding had the optimum mechanical properties after quenching at 1180 °C and triple tempering at 540 °C, with a hardness of 64.2 HRC and a flexural strength of 2858 MPa, much higher than the casting alloy with a similar composition. Luo et al. [20] found that the tempering time has a certain effect on the carbide properties and growth; the appropriate holding time can produce fine secondary carbides and smaller grain size, thus effectively improving the impact toughness and wear resistance.

W-Mo-V high-speed steel is a new type of high-carbon, high tungsten, high-molybdenum, high-vanadium, high-chromium, high-hardenability tool steel; however, there have been few studies on the use of this steel for the preparation of high-speed steel rolls, and there is no accurate description of its chemical composition, heat-treatment-process parameters (annealing, quenching and tempering temperatures) and working environment. In this paper, JMatPro software was used to simulate and predict the performance changes of W-Mo-V high-speed steel under different alloying-element ratios, and the simulation data were used to adjust the alloy content and heat-treatment-process parameters of high-speed steel. Subsequently, according to the heat-treatment-process parameters designed in this paper, centrifugal casting, combined with metallurgical technology, was used to prepare HSS samples for heat treatment. Finally, the samples were compared with the properties of conventional HSS rolls. The microstructures were compared and observed to see if the microstructure and grain size were improved. If they are, this study will help to extend the service life of W-Mo-V HSS mill roll coverings, improve productivity and reduce production costs.

## 2. Materials and Methods

### 2.1. Materials

According to the literature [1,12,16,21,22], to determine the study of the W-Mo-V system of high-speed steel-element content range (shown in Table 1), the experimental material for commercial high-speed steel powder, is that of the composition shown in Table 2. Based on maintaining the basic composition, it is adjusted and certain amount of other trace elements are added, to improve the mechanical properties; that is, several groups are designed to determine the content of the alloying element of high-speed steel, and the JMatPro software is used for preliminary analysis.

### 2.2. Research Program

The subject of this research is the effect of various alloying elements such as W, Mo, and V on the performance of W-Mo-V HSS and the change in the role of the microstructure, in order to be able to design suitable materials according to the working conditions of high-speed steel rolling-mill roll sets, and preferring the economically reasonable hot-processing route. This research includes the following points:

(1) The use of empirical formulas and a literature discussion, theoretically determining the alloy composition of W-Mo-V system high-speed steel.
(2) Determining the range of the content of each element of W-Mo-V high-speed steel, in which, based on maintaining the basic composition, it is adjusted and a certain amount of other trace elements are added to optimize its mechanical properties, wear resistance, thermal hardness, and corrosion resistance, etc.; i.e., the design of many groups of high-speed steels with a determined content of alloying elements is analyzed by using the JMatPro software, and finally, a set of data is optimized and selected for the best set of material properties.
(3) JMatPro software is used to carry out thermodynamic phase calculations, alloy-composition phase diagram analysis, solidification calculations, mechanical property calculations, high-temperature strength calculations, and the output of TTT/CCT diagrams, and to analyze the experimental data, to design a reasonable heat treatment process, to achieve the purpose of regulating the material’s organizational structure, refining the grain, and improving the strength and toughness of the material.
(4) Using the JMatPro (version 7.0) simulation software, the solidification process and phase-change law of the W-Mo-V system high-speed steel were analyzed. At the same time, the effects of annealing temperature, quenching temperature, and tempering temperature on the organization and properties were analyzed to determine the optimal range of annealing temperature, quenching temperature, and tempering temperature for W-Mo-V high-speed steel. It provides an important theoretical basis for the selection of heat-treatment-process parameters for these types of materials.
(5) General conclusions are drawn from the virtual fabrication of the W-Mo-V HSS mill-roll-cover material, and the results are summarized and discussed to determine the suitable chemical composition and heat treatment process parameters. Finally, the use of a vertical centrifugal casting machine to make samples is examined, and a suitable heat treatment process is carried out. The microstructure of the samples are then observed using a Quanta 250FEG scanning electron microscope (SEM) (FEI, New York, NY, USA).

## 3. W-Mo-V System of High-Speed Steel Composition Selection

### 3.1. Determination of the Content of W, Mo, V, and Cr Elements

Tungsten, molybdenum, vanadium, and chromium elements are extremely important alloying elements in high-speed steel. The importance of the alloying elements in high-speed steel lies in their influence on the type of carbides formed and the formation temperature, as well as their influence on martensitic tempering. The matrix of high-speed steel rolls mainly includes MC, M_2_C, M_6_C, M_7_C_3_, M_23_C_6_ and other carbides [2,9,23,24], and these carbides are the key factor in improving the wear resistance and impact resistance of steel, while the presence of these carbides can also greatly improve the effect of subsequent heat treatment of HSS.

Tungsten mainly forms tungsten carbide (WC) in HSS, which mainly forms M_6_C-type carbides. The addition of tungsten can improve the comprehensive mechanical properties of HSS rolls, such as wear resistance, hot hardness, strength and toughness, etc. The dissolution of M_6_C carbide in austenite can promote a significant increase of carbon and other alloying elements in the austenite, thus stabilizing the austenite phase and improving the hardness and thermal stability. At the same time, at high temperatures, M_6_C and MC composite carbides will undergo eutectic reactions and form M_2_C carbides, which are rich in W, Mo, and V can promote the formation of M_6_C carbides [25]. During heat treatment, in quenching, the addition of tungsten prevents overheating of HSS, thus reducing the formation of a decarburization layer in HSS. During tempering, W is solidly dissolved in martensite, thus strengthening the martensitic phase, and also precipitates as secondary carbides, resulting in secondary hardening. It has been shown [26] that the addition of WC leads to a denser microstructure of HSS and reduces tissue defects and porosity, and no porosity was observed when the WC content reached more than 5%. However, it is not the case that the higher the content of tungsten, the better the performance of HSS; the appropriate amount of WC can improve the comprehensive mechanical properties of the material, and if the content is too high it will destroy the continuity of the matrix, so that the flexural strength and impact toughness is significantly reduced. Content within 10% of its strength and the toughness change show first a higher and then lower trend: 5% for the peak, combined with a commercial HSS powder W content of 5.8%. That is, in the determined range, the tungsten content was determined to be 5.5%.

Molybdenum in high-speed steel mainly forms molybdenum carbide (MoC). Because tungsten and molybdenum are both strong carbide-forming elements, their essential properties are identical [5]. Mo will first form a suitable M_2_C-type carbide, and in the subsequent thermal processing, the sub-stable M_2_C-type carbide will be decomposed, resulting in the formation of a fine, stable MC and M_6_C-type carbide. Compared with W, the mass fraction of Mo is about one-half of W, and the solubility of molybdenum in austenite is about 1.45 times higher than that of tungsten [25]. Its distribution in steel will be more uniform; i.e., Mo can be one-half of the amount of W to give the same effect on HSS as that of W. That is, Mo can give the same effect on HSS as W, in half the amount of W, but increasing the molybdenum content on top of that will result in the formation of more fine, stable MC and M_6_C carbides in HSS. However, excess molybdenum increases the brittleness of HSS and reduces its impact strength and toughness. Therefore, it was decided to retain the molybdenum content of 5.0% in commercial HSS powders.

Vanadium is mainly formed in HSS as vanadium carbide (VC), which mainly forms MC-type carbides. The inclusion of vanadium increases the hardness and wear resistance of HSS [2]. The inclusion of a high number of hard MC and M_2_C carbides into the HSS roll organization can significantly increase the roll’s wear resistance, extending its service life [27]. At the same time, V has a refining effect on the HSS matrix organization, which can further improve the strength and toughness of HSS. With the increase in vanadium content, the number of MC-type carbides is greater, and the strength and wear resistance are increased. And V, N and O elements have a greater affinity, and VC dispersion is high and very stable; it is conducive to deoxidation and achieves dense, fine grain organization, thus improving plasticity, toughness, and strength. Its V content is between 4.0 and 6.0%; high-speed steel wear resistance tends to increase and then decrease, but the change is small [27]. However, excessive vanadium can crack grain boundaries, thus reducing their wear and corrosion resistance. That is, it was decided to take a vanadium content of 5.5% within the specified range.

Chromium in high-speed steel mainly forms chromium carbide (Cr_3_C_2_), which mainly forms M_23_C_6_-type carbides. The inclusion of chromium improves steel’s wear resistance and hardenability, while also improving corrosion and oxidation resistance [26]. Cr belongs to the medium carbide-formation elements, the formation of carbides is fine and uniform, and in the quenching process, most of the fine M_23_C_6_ can be dissolved in the austenite matrix, thus refining the grain, improving the toughness of high-speed steel and reducing the deformation effect of quenching. It has been shown [27] that chromium (Cr) and molybdenum (Mo) content of a balanced increase can effectively improve the hardness of high-speed steel rolls. A certain content of Cr can promote the precipitation of secondary carbides, thus enhancing the secondary hardening ability. When the chromium content is too high, on the contrary, it will be detrimental to the tempering stability of martensite and red hardness. In this paper, we study the mill roll-cover material for high strength and toughness of high-speed steel, with a chromium content of 4% to 5%. To summarize, the chromium content is determined as 4.5%.

### 3.2. Determination of Other Alloying Elements

In addition to tungsten, molybdenum, vanadium, and chromium, the four most important alloying elements, we will add a small number of other alloying elements to further improve the high-speed steel rolls, such as niobium (Nb), silicon (Si), manganese (Mn), boron (B) and other elements. These elements, like the major elements mentioned above, can be combined with carbon to form carbides.

The combination of niobium and carbon in steel forms stable carbides and grain refinement, which improves the strength and hardness of HSS mill roll covers. In high-speed steel, Nb and C combined to form a reticulate MC-type carbide; the carbide can promote other elements’ better solid solution in the matrix, to improve the secondary hardening capacity, and the formation of niobium carbide (NbC) can be used as austenite nucleation points, to promote the austenite non-homogeneous nucleation, so that it produces more grains, and thus a refinement and homogeneity of the austenitic organization [3]. High-speed steel, vanadium, and carbon-binding capacity are similar, and when vanadium and carbon are combined with the formation of more MC-type carbides, the liquid phase will precipitate more grains, and its role is the same as niobium. In this paper, the study shows that if the high-speed steel rolls’ roller-sleeve material V content is higher, and if the Nb content is also designed to be higher, it will lead to too many MC-type carbides, so that the liquid phase accounted for a relatively large proportion of the weakening of other grains on the growth of its interference, which leads to the growth of MC-type carbides, which become longer, and lead to coarse grains. Therefore, the niobium content in the determined range of 0.5% can be taken.

Silicon will combine with oxygen in HSS, and the oxide it forms will be tightly and firmly bonded with the grain cells at the metal boundary, and will not come off even at high temperatures, which greatly improves the heat resistance and wear resistance of HSS. Silicon is also effective in slowing down the decomposition of tempered martensite at low temperatures, and it has a weak inhibiting effect on grain growth [28]. But it is also in the form of silicate in the HSS, thus reducing the performance of HSS, so the silicon content should not be too much, to determine that the range of 0.7% can be taken.

The addition of manganese can improve the hardness and thermal stability of HSS, and it can reduce the deformation that occurs during quenching [29]. Manganese, as an alloying element, not only cannot inhibit the growth of austenite grains, but also may cause coarse crystals, although the presence of trace amounts of manganese can promote carburization and improve the surface hardness of HSS rolls. In summary, the manganese content is taken as 0.55% in the determined range.

Boron and nitrogen belong to low-cost alloying elements, and B and N added in the right amount can be done to improve the performance of high-speed steel rolls at low cost. Boron and niobium, together, at grain boundaries form an M_3_B_2_ phase, thereby increasing the strength of the steel grain boundaries, as well as their creep resistance, and when boride-containing alloys and carbide-containing alloys have the same combination of hardness, the hardness is higher than carbide-containing alloys [30]. The addition of trace nitrogen can make the carbide distribution in high-speed steel more uniform, and improve the impact toughness [31]. Kengo Yoshimoto [16] and others showed that boron (B) and nitrogen (N) can be used in the addition of alloying elements, for the solidification of high-speed steel roll organization and the hardness of the impact, and found that a certain amount of the addition of B and N can improve the hardness of high-speed steel. Therefore, it was determined that the boron content should be taken as 0.2% and the nitrogen content as 0.06%.

### 3.3. Determination of (C) Carbon Content

Carbon is the most essential alloying element in HSS, and a solid solution of carbon and iron may generate solid-solution strengthening, which reduces dislocation movement and increases HSS strength and hardness. Carbon content determines the nature of the high-speed steel matrix [32]; carbon, in the preparation of high-speed steel of each process, also has a great impact, because it can be almost high-speed steel in all the elements of the formation of carbides, and these carbides greatly affect the mechanical properties of high-speed steel and the performance after heat treatment.

High-speed steel rolls are used for the general carbon content greater than 1.5% of high-carbon high-speed steel. With high-carbon steel in the quenching, part of the carbon will be dissolved into the austenite to improve the strength of martensite, and the tempering; part of the alloying elements of the carbides will be dispersed in precipitation, resulting in the formation of the secondary hardening, and the organization of grain growth, and ultimately in the improvement of the wear resistance of high-speed steel, red hardness, and hardness [33]. When the carbon content is too low, the transformation of the appeal phase is difficult to complete, so the prepared high-speed steel does not achieve the expected excellent performance. On the contrary, too high a carbon content will produce more residual austenite after quenching, which will make the steel very susceptible to deformation during the cooling process, and at the same time, it will make the steel surface easy to wear, greatly reducing its wear resistance [34]. In short, carbon exists in steel, and with the increase of carbon content in austenite, carbon, in the promotion of austenite-grain growth in elements, will promote the growth of austenite grain. However, if insoluble carbide exists in steel, will inhibit grain growth, thus playing the role of grain refinement, and the austenite in high-speed steel grain size will directly affect the high-speed steel after cooling and its organization and properties [5].

Carbon in HSS is expected to exist in the form of carbides, so the determination of carbon content needs to be based on the content of other alloying elements that have been determined to achieve “carbon balance”. The carbon balance formula (Equation (1)) [1] initially determined that the carbon content was 1.8665%, but this is only based on empirical formulas, and does not represent the carbon content in the actual improvement of the performance of high-speed steel rolls the best, and therefore we need to use the JMatPro software to simulate the different carbon content, to take the best value. In the determined range, every 0.1% takes a set of data, and the results are shown in Table 3.

ω_c,e_ = 0.06Cr + 0.033W + 0.06Mo + 0.2V (wt.%) (1)

The organization of HSS mainly includes carbide, martensite, bainite, and residual austenite. One of the carbides has a high hardness of high-speed steel within the organization; its hardness and wear resistance have a huge impact, and determine that the hardness of high-speed steel organization is mainly a martensitic organization. The residual austenite and bainite organization of high-speed steel toughness and impact resistance have a very important impact. Therefore, to control the performance of high-speed steel, a large part of the high-speed steel controls the performance of the martensite organization. The martensite organization is in the cooling process, from the austenite transformation, so the performance of the austenite organization in the high-temperature state is also a large part of the decision of the performance of the martensite organization. According to the literature [35,36,37], martensite is a supersaturated solid solution, and a high-speed steel from the state of austenite to a very fast rate of cooling to M_s_ (martensite transformation-onset temperature) from below the beginning of the non-diffusive martensite transformation until the M_f_ transformation, will result. After the austenite is cooled below M_f_ to end the martensitic transformation, a portion of the austenite remains untransformed, and this austenite becomes residual austenite. Therefore, how much martensite can be transformed depends on the M_f_; however, the higher the carbon content in the austenite, the lower the M_f_ will be, and the more residual austenite there will be, with the increase in the content of residual austenite making the strength and toughness of high-speed steel decline, therefore minimizing the content of the residual austenite.

**Figure 1:**
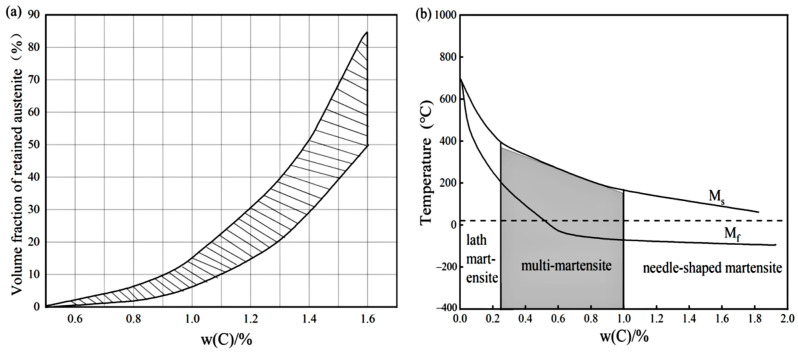
(**a**) Effect of carbon content in austenite on the amount of residual austenite; (**b**) effect of carbon content in austenite on martensitic transformation temperature [37].

According to the literature [37], in a certain range, with the increase of carbon content in the austenite, the martensitic transformation temperature and the end of the temperature are gradually reduced, while the content of residual austenite gradually increases, as shown in Figure 1. From the curve in Figure 1a, when the carbon content in austenite rises to 0.6%, when the martensitic transformation termination temperature is closest to room temperature, and from Figure 1b, when the carbon content in austenite is below 0.7%, the residual austenite content is lower. Martensite organization contains lath and lamellar martensite; lath martensite has better plasticity and toughness, and the higher the carbon content, the better the performance of the lath martensite in reducing the performance of poorer lamellar martensite, so we take the austenite carbon content of 0.6% to 0.7% to be the best.

In conclusion, the carbon content of 1.9% is consistent with the formation of optimal martensitic organization. Subsequently, we continued to use JMatPro software to simulate and calculate the CCT curve for a certain temperature range to observe the martensite transformation temperature, thus further determining the optimal carbon content.

From the data in Table 4,it can be seen that, with the increase in temperature, the austenite content gradually reached the peak, and the martensite began to transform. The temperature appears to first decrease and then increase, and then to reduce the trend. M50 and M90 also show the same trend. When the quenching temperature reaches 1150~1160 °C, M90 is closest to room temperature, so the carbon content of 1.9% is reasonable.

Combined with JMatPro software simulation calculations, and taking into account the fact that the high-speed steel rolls need to have a variety of properties, as well as the working environment and the impact of each alloying element on the organization and performance of high-speed steel, together with the interactions between the various alloying elements, we ultimately came up with the W-Mo-V system of high-speed steel rolling-mill roll-cover material for each alloying element content, as shown in Table 5.

## 4. W-Mo-V High-Speed Steel-Phase Diagram Analysis and Heat-Treatment-Process Design

### 4.1. W-Mo-V High-Speed Steel-Phase Diagram Analysis

**Figure 2:**
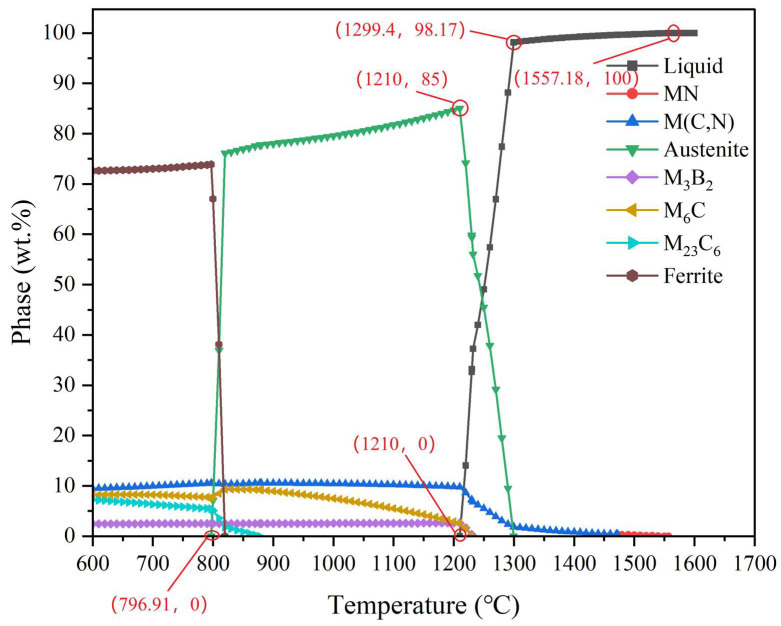
Equilibrium-phase diagrams of Fe-5.5W-5.0Mo-5.5V-4.5Cr-0.7Si-0.55Mn-0.5Nb-0.2B-0.06N-1.9C high-speed steels at different temperatures (the numbers indicate weight percent).

Figure 2 shows the equilibrium-phase diagram of HSS at different temperatures, calculated by simulation using JMatPro software and selecting Step Temperature in General Steel. Set the temperature range of 600~1600 °C, in which every 10 °C is calculated at this time for the content of each phase and the content of each element in the phase, and additionally calculate the content of each phase of the most value and the most value for each alloying element in the phase.

As can be seen in Figure 2, the HSS undergoes three phase zones as a function of temperature, namely, the ferrite, austenite, and liquid phases. It also contains four carbides and an M_3_B_2_ phase, consisting mainly of niobium and boron, which are MN, M (C, N), M_6_C, and M_23_C_6_, respectively. The ferrite zone is present in the range from 600 °C to 796.91 °C, since austenite starts to appear from 796.91 °C. The austenite zone is also present in this temperature range. Four other phases, M (C, N), M_6_C, M_23_C_6_, and M_3_B_2_, are present in this temperature range. With the gradual increase in temperature to 796.91 °C, ferrite will be all dissolved in austenite, and with the gradual increase in temperature, M (C, N) and M_3_B_2_ mass percentage will have a tendency to increase. However, the change is not large, and the M_3_B_2_ change is weak; M_6_C mass percentage will have a small first increase and then decline within the trend, and the M_23_C_6_ has a significant decrease in the trend. The phase will also have a significant decrease in the temperature of the austenite, and the phase will also disappear gradually, as the temperature continues to become higher. From 796.91 °C to 1220 °C is the austenite region, and at 1210 °C it reaches the austenite peak; M_3_B_2_ still shows a weak rising trend, but when the austenite content reaches the peak it will fall sharply, until disappearance. M (C, N) and M_6_C both show a brief rise and then fall, which for M (C, N), due to the large number of high melting points and high hardness of VC, makes its melting point higher, and M_23_C_6_ has a significant decrease trend, and this phase will also disappear with the continued high temperature. M (C, N) has a high melting point, due to the large amount of high melting points and the high hardness of VC, so it shows a slow downward trend and a sudden drop occurs after exceeding the austenite peak, at which time the liquid phase will also begin to appear. M_6_C, on the other hand, has a relatively low melting point, and its mass percentage has been plunging. By about 1230 °C, the M_6_C and M_3_B_2_ phases are completely dissolved. At 1471.96 °C, M (C, N) is completely dissolved, and at this time the MN phase begins to appear; the MN phase with the vanadium and nitrogen elements is higher, so its melting point is higher than the VC, but then the content will also be reduced with the increase in temperature until, at 1557.18 °C, it is completely dissolved, the alloy is completely in the liquid phase, and, with the drop in temperature, the liquid phase will precipitate solids. These solids include the MN phase, M_6_C phase, M (C, N) phase, M_3_B_2_ phase, and an austenite phase; when the temperature drops to 1472.19 °C, the liquid phase will result, and the MN has a common role in the occurrence of encapsulated crystal transition, thus generating M (C, N), and the MN completely disappears. When the temperature reaches 1210 °C near, this time the austenitization reaches the maximum degree, and the liquid phase also almost disappears; in this temperature interval, the alloy is in the solid–liquid coexistence state.

### 4.2. Analysis of Carbides in W-Mo-V High-Speed Steels

#### 4.2.1. Analysis of Each Alloying Element in Ferrite

**Figure 3:**
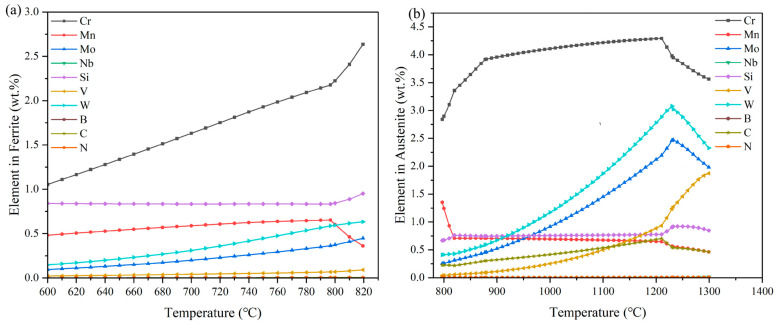
Analysis of elemental content in HSS Fe-5.5W-5.0Mo-5.5V-4.5Cr-0.7Si-0.55Mn-0.5Nb-0.2B-0.06N-1.9C: (**a**) elemental content in ferrite; (**b**) elemental content in austenite.

As shown in Figure 2, the ferrite phase region is the first phase region experienced by HSS in the process of heating and melting. Using JMatPro software simulation, the calculation of the ferrite phase region of the alloying elements, with the temperature changes in the process, is shown in Figure 3.

The main element in ferrite is Fe, and Figure 3a demonstrates the mass percentage change of each alloying element. It is clear from the figure that the content of the Cr element is increasing with the increase in temperature, and W and V elements also show a weak increasing trend. Combined with Figure 2, it can be seen that Mn and Si are in a relatively stable state before the ferrite content reaches the peak, and these two elements play a role in stabilizing the ferrite. After the peak of ferrite, Si tends to rise abruptly, while Mn decreases abruptly. V, N, C, and B are trace elements with low content and little change. In the ferrite phase region, due to the ferrite dissolved in the carbon being very low, this time the carbon element is mainly combined with other strong carbide elements to form several high melting points and high-hardness carbides, such as M_6_C and M_23_C_6_, while the B element and niobium combined to form the M_3_B_2_ phase.

#### 4.2.2. Analysis of Each Alloying Element in Austenite

As shown in Figure 2, the austenite phase region is a phase region following the ferrite phase region, and starting from 796.97 °C, the ferrite phase starts to undergo austenitization, thus transforming into austenite. Figure 3b shows the variation of each alloying element in austenite with the temperature, as calculated using the software simulation. The main element of austenite as the dominant phase in this temperature interval is iron. As shown in Figure 2, the austenite content is plunging before 820 °C; i.e., the ferrite plunges. At this time, ferrite austenitization, in this temperature interval before the ferrite contains the elements, the austenite equivalent is contained. As shown in Figure 3b, before the austenite content reaches its peak, the element Cr is rising, and due to the presence of the strong carbide Cr element, the carbon in the austenite also shows a rising trend. However, after the austenite reaches the peak of the austenite, which has occurred after a sudden decline in the Mn element content, it will initially have a sudden decline in the trend, and later content will be similar to that of the Si element. This will always maintain a more stable state, and, due to Mn and Si’s simultaneous existence, they work together, and can play a role in inhibiting the austenite growth, to refine the austenite grain effect. W, Mo, V elements in the austenite reached the peak before there had been W, Mo, V elements in the austenite; before reaching the peak, here had been in a rising trend. For the austenite content to reach the peak, there will be a sudden change. The M and Mo content plummeted, while the V content surged, because the V formation of carbide ability is strong, and VC belongs to a high melting point of the high hardness of the carbide. In the austenite phase region, the Nb and B content in the austenite content is small, but they work together to improve the hardness of austenite. After the austenite reaches its peak, the alloy begins to appear in the liquid phase and the alloying elements in the austenite begin to change abruptly.

The austenite phase region is extremely important in the alloy, which should be used for the subsequent heat treatment. The principle is to heat the alloy to the austenite phase region, and then by changing the austenite organization and making it undergo a martensitic transformation during cooling, to obtain a martensitic organization with excellent properties. Therefore, the properties of the austenitic organization greatly influence the final properties of HSS. The alloy content in the austenite will directly affect the austenitic organization, high alloy content helps it contain a higher content of high hardness and high melting point carbides, and some alloys also help to refine the austenitic grain, thus making the heat treatment of the martensite organization grain is more fine, uniform, thus improving the performance of high-speed steel in all aspects.

#### 4.2.3. Analysis of the Alloy Content of the Remaining Four Carbides

These four carbides include MN, M (C, N), M_6_C, and M_23_C_6_. These four carbides are mainly composed of alloying elements, as shown in Figure 2; the content of these four carbides in the entire phase diagram is not high, but can greatly affect the final performance of high-speed steel.

**Figure 4:**
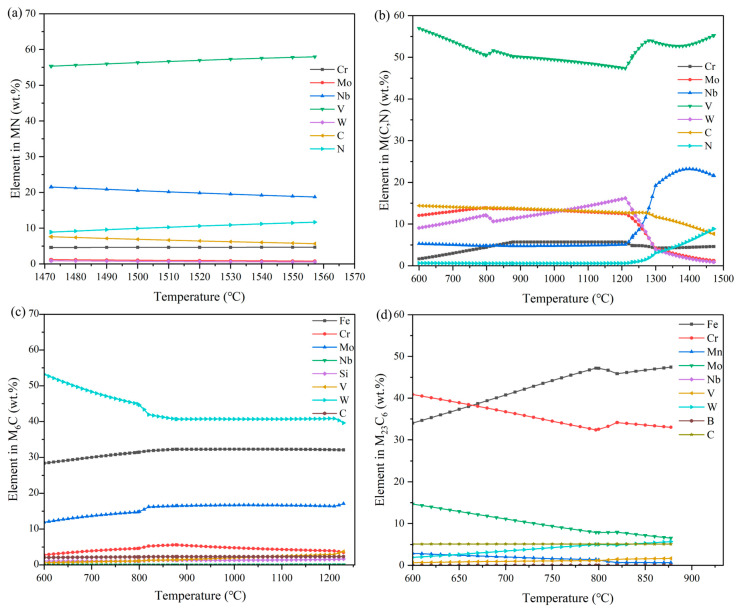
Analysis of the content of different carbides in Fe-5.5W-5.0Mo-5.5V-4.5Cr-0.7Si-0.55Mn-0.5Nb-0.2B-0.06N-1.9C high-speed steel. (**a**) Elemental content in MN; (**b**) elemental content in M (C, N); (**c**) elemental content in M_6_C; (**d**) elemental content in M_23_C_6_.

As shown in Figure 4a, the carbide main alloying elements and V, and vanadium formed by the carbide melting point are higher; combined with Figure 2,it can also be seen that the carbide with the increase in temperature is the last melting solid phase. Overall, the content of alloying elements in MN does not vary much, and there is stabilization.

As shown in Figure 4b, this carbide also contains mainly V elements, and all these elements will appear to rise or fall abruptly in the region of austenitization and austenitization dissolution. At the same time, it can be seen that the carbide does not contain silicon and manganese elements, and in the high-temperature state, the VC content is high, Nb will appear as a rising change, and the content is not low.

From Figure 4c, combined with Figure 2, it can be seen that after austenitization, the contents of all elements are more stable, but there will be some M_6_C transformed to austenite in the process of austenitization. The carbide is mainly W. The content of Fe is about twice as much as that of Mo, and the content of both is not high. The content of Cr, C, Si, and V is low, and not much changed. Tungsten carbide and molybdenum carbide are the main elements of M_6_C, which can refine the austenite grains, and at the same time, improve its thermal stability.

Figure 4d shows that M_23_C_6_ carbide mainly contains Fe and Cr elements, and the content of the two shows an opposite trend to change; combined with Figure 2, it shows that M_23_C_6_ carbide mainly exists in the ferrite, and with the austenitization of ferrite, part of the M_23_C_6_ will be encapsulated with ferrite to produce M_6_C; the other part dissolves in the austenite, and M_23_C_6_ will be dissolved in the austenite. M_23_C_6_ is mainly in tungsten carbide and molybdenum carbide. Austenite, the M_23_C_6_ in the austenite mainly in the form of particles, and its subsequent tempering, promote secondary hardening.

#### 4.2.4. Analysis of Alloying Elements in M_3_B_2_ Borides

**Figure 5:**
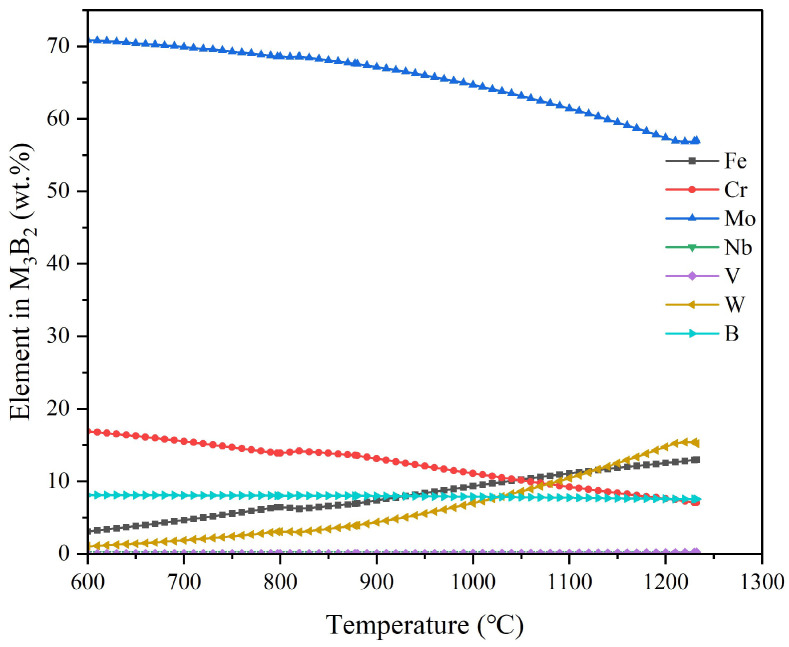
Analysis of elemental content in M_3_B_2_ borides in Fe-5.5, W-5.0Mo-5.5V-4.5Cr-0.7Si-0.55Mn-0.5Nb-0.2B-0.06N-1.9C high-speed steel.

Figure 5 is the M_3_B_2_ boride alloying elements with the temperature changes. As can be seen from the figure, it mainly contains Mo elements, and with the high temperature of the body it presents a slight downward trend, for which the Cr element is also the same. The Fe element and the W element content is similar, and presents a slow upward trend, while the B element and the Nb element content is the same, together with the stable existence of the boride. The stable existence of the boride improves the hardness of HSS, to a certain extent.

### 4.3. Determination of Heat-Treatment-Process Parameters

#### 4.3.1. Determination of Annealing Temperature

**Figure 6:**
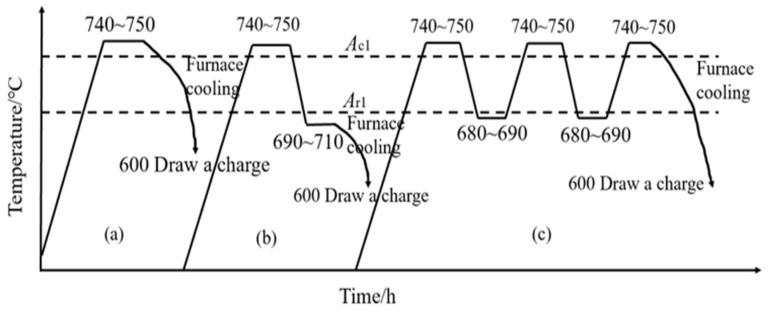
Schematic diagram of several spheroidal annealing processes for alloy steels.

The annealing process is also known as preparatory heat treatment; its main purpose is to adjust the organization and properties of steel parts, to eliminate defects in the metallurgical and thermal processing of steel, and to facilitate the subsequent mechanical processing and heat treatment. Annealing is carried out to heat the steel to the A_c1_ temperature near the holding time, followed by slow cooling to obtain a more balanced organization. For over-eutectic steel and high-alloy steel generally, we use spheroidal annealing; spheroidal annealing can be to transform the high-speed steel carbide into spherical or granular, and to be evenly distributed in the ferrite matrix. This can achieve uniform organization, reduce the hardness, and improve the machinability of the purpose, at the same time, in order for the subsequent quenching to do a good job on the organization of the preparation. The spheroidal annealing temperature is generally taken to be higher than the A_c1_ temperature of 20–30 °C. Through the use of the JMatPro software simulation calculations of the phase equilibrium diagram that can be seen in this paper, the design of the W-Mo-V system of high-speed steel, A_c1_ is 796.91 °C, and A_ccm_ is 819.49 °C. Therefore, the annealing temperature is determined as 817–827 °C. Its specific spheroidal annealing process can be referred to in Figure 6 [38].

#### 4.3.2. Determination of Quenching Temperature

A suitable quenching process can significantly improve the strength and hardness of HSS, and at the same time, to make it also have a certain degree of plasticity and toughness, it is necessary to temper it after quenching, to eliminate its residual internal stresses [37].

Quenching is the process of heating the steel above the A_c1_ or A_c3_ temperature at a certain rate, holding it for a certain period, and then cooling it at a rate faster than the critical cooling rate, to obtain a martensitic or bainitic organization with excellent properties. The W-Mo-V high-speed steel designed for this study is a peritectic, high-alloy steel. For over-eutectic steel, its quenching is usually heated to A_c1_ above 30–50 °C; the purpose is to obtain the fine-grain austenite, while also retaining a certain amount of high-hardness insoluble carbide, thus making the martensite transformation after the martensite grains are also finer, to improve the comprehensive mechanical properties of high-speed steel. If the quenching temperature is too high, that is, more than the A_ccm_ temperature, the austenite will incorporate more carbides, so that the carbon content increases, resulting in M_s_ being lower, residual austenite increasing, and hardness and wear resistance is being reduced. However, the design of high-speed steel is a high-alloyed steel, which contains a large number of strong carbide elements, with the need to make the full alloying, the need for higher quenching temperatures, and for the quenching temperature of up to 200–500 °C above A_c1_.

As the high-speed steel belongs to the high-carbon high-alloy steel, its quenching temperature can be taken to be above A_c1_ 200–500 °C. In this temperature range, as reference to Figure 2 shows, in addition to austenite there also exist M (C, N), M_6_C, and M_3_B_2_, of which M (C, N) and M_3_B_2_ content is more stable, with fine-grain distribution uniformity, and excellent performance. The M_6_C-type carbide is fine lath or fishbone, and will make the austenite in the subsequent martensitic transformation give a poor performance of lamellar martensite, so we choose the M_6_C content of low temperature as the quenching temperature; that is, the Figure 2 selection of the quenching temperature of 1130~1230 °C.

**Figure 7:**
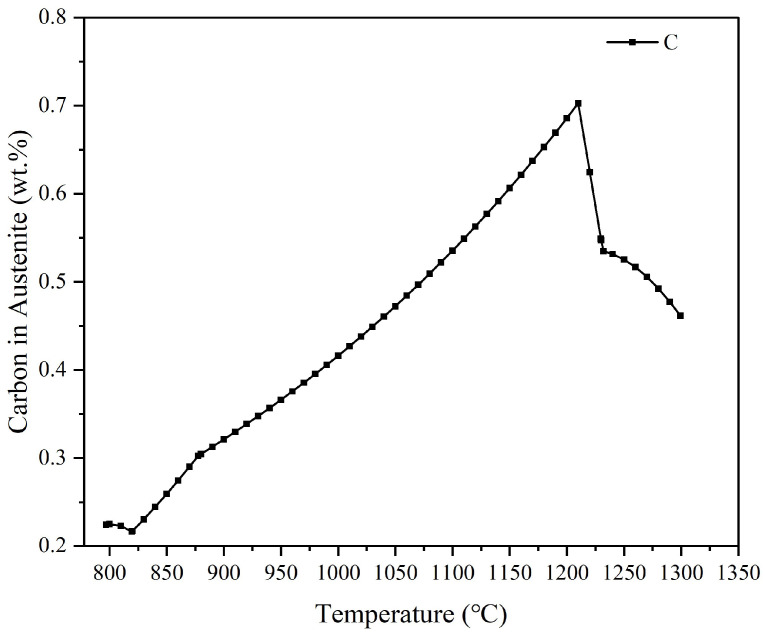
Carbon content of austenite at different temperatures.

The carbon content of the austenite also greatly influences the properties of the martensitic organization that arrives after cooling and after quenching. Figure 7 shows the carbon content of the austenitic organization at different temperatures. As can be seen from Figure 1, when the carbon content in the austenite is between 0.6 and 0.7%, when the residual austenite content is lower, martensite has better plasticity and toughness. That is, it is combined with the Figure 7 analysis of the quenching temperature taken for 1150~1200 °C. According to the data in Table 4, if the quenching temperature is in the range of 1150~1160 °C, the temperature of M90 is closest to room temperature. Therefore, it is determined that the quenching temperature range of W-Mo-V high-speed steel designed in this paper is 1150–1160 °C.

#### 4.3.3. Determination of Tempering Temperature

A suitable tempering process can effectively reduce or eliminate the residual internal stresses generated during quenching, thus achieving the effect of toughening, and by adjusting the tempering temperature to the desired organizational transformation, obtain the strength, hardness, and plasticity of the appropriate fit [39].

Tempering refers to the heating of quenched steel at temperatures below A_1_ to obtain a stable organization and then cooling them in a certain way to obtain tempered martensite, tempered to site, or tempered sostenite organization, with excellent properties. Tempering can be divided into low-temperature tempering, Chinese tempering, and high-temperature tempering. For W-Mo-V high-speed steel, we generally use high-temperature tempering. The temperature of high-temperature tempering is generally between 500 °C and 650 °C, for high-speed steel after high-temperature tempering, due to its strong anti-tempering properties, so that its tempered organization is still martensite, so it can produce the effect of secondary hardening [40]. The A_c1_ temperature of the W-Mo-V series high-speed steel designed this time is approximately 800 °C. The tempering temperature is lower than the A1 temperature. The quenching temperature here is 1155 °C, and the tempering temperatures are 760 °C, 730 °C, 700 °C, 670 °C, 640 °C, 610 °C, 580 °C, and 550 °C, respectively. For the temperature, we use simultaneous precipitation in JMatPro software to calculate the changes in carbides and grain size that will precipitate in the high-speed steel during the tempering process, with time.

**Figure 8:**
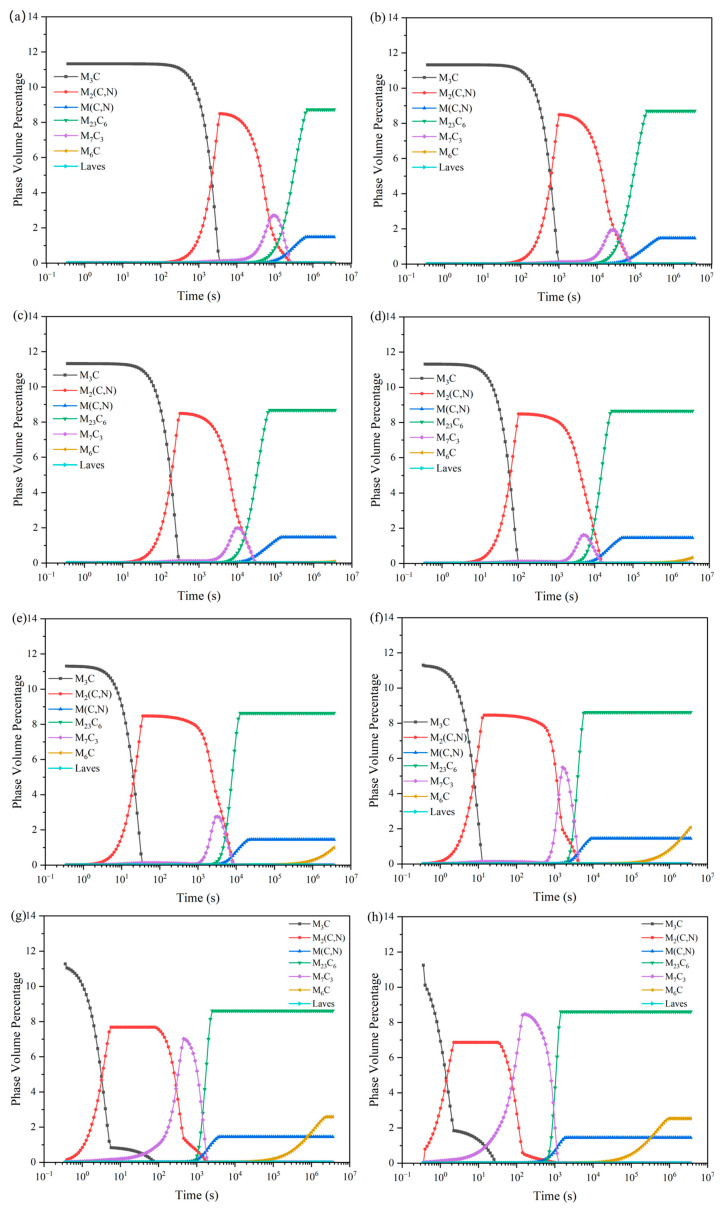
Fe-5.5W-5.0Mo-5.5V-4.5Cr-0.7Si-0.55Mn-0.5Nb-0.2B-0.06N-1.9C. Variation of carbide content of each carbide with the tempering time at different tempering temperatures of high-speed steels: (**a**) 550 °C; (**b**) 580 °C; (**c**) 610 °C; (**d**) 640 °C; (**e**) 670 °C; (**f**) 700 °C; (**g**) 730 °C; (**h**) 760 °C.

**Figure 9:**
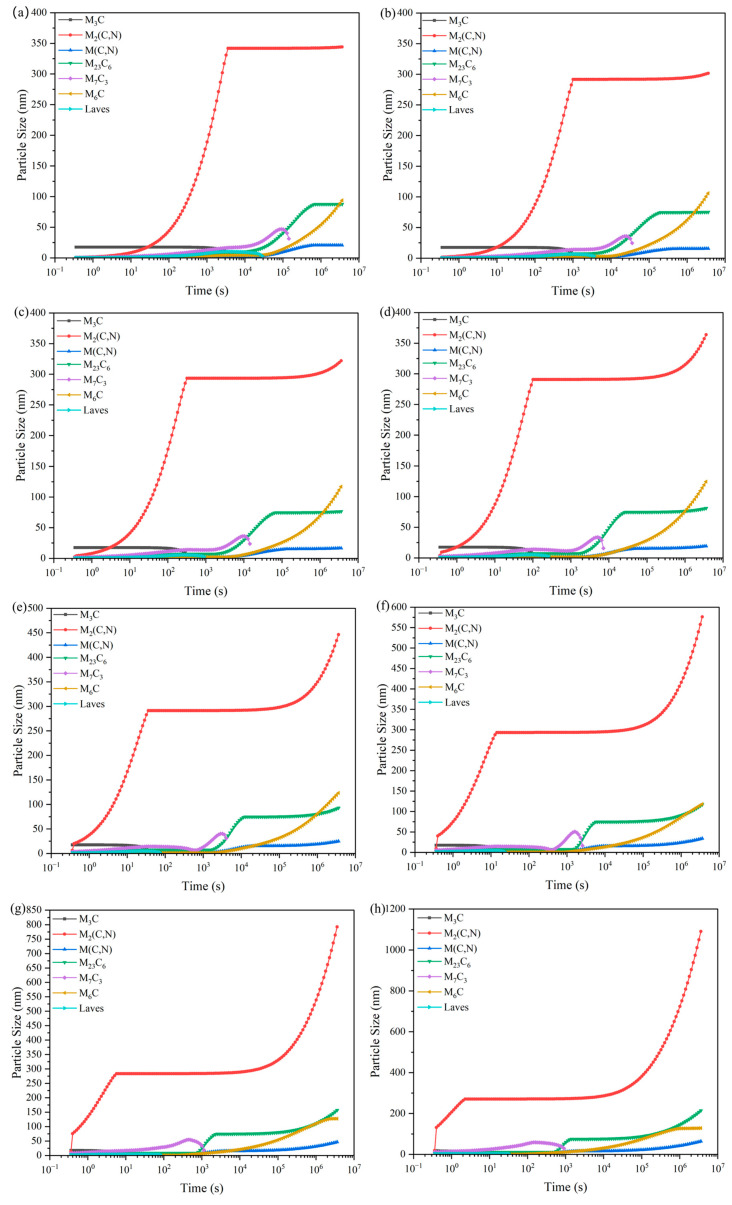
Fe-5.5W-5.0Mo-5.5V-4.5Cr-0.7Si-0.55Mn-0.5Nb-0.2B-0.06N-1.9C. Variation of carbide grain size with tempering time in high-speed steels at different tempering temperatures: (**a**) 550 °C; (**b**) 580 °C; (**c**) 610 °C; (**d**) 640 °C; (**e**) 670 °C; (**f**) 700 °C; (**g**) 730 °C; (**h**) 760 °C.

As can be seen from Figure 8, with the increase in tempering temperature, the M_3_C, M_2_ (C, N), M (C, N), M_23_C_6_ carbide peak content is unchanged, and the M_7_C_3_ peak appeared to show a clear trend of first declining and then rising. At 550~640 °C, stabilization exists within 2 h. And, in a certain time range, the higher the tempering temperature, the more the M_6_C carbide content is also rising. The presence of M_7_C_3_ carbides can favorably improve the high-temperature resistance, impact resistance, and wear resistance of HSS. And, because the M_7_C_3_ carbide in Figure 9 shows that after tempering for two hours, its grain size will have a tendency to increase with the increase in temperature, it is not conducive to improving the performance of HSS rolls. In summary, in about 2 h within the tempering holding time, the best tempering temperature selection is 550–610 °C.

## 5. W-Mo-V High-Speed Steel Experimental Verification Analysis

According to the alloy composition of the W-Mo-V system HSS designed in the previous section, due to the high content of its alloy composition, care should be taken to reduce or prevent the segregation of alloying elements when preparing the samples. Although all aspects of this HSS were simulated and calculated using JMatPro software, which showed better performance compared to the existing HSS roll materials, the actual production application will be affected by many other environmental factors. Therefore, it is necessary to prepare samples for experiments to make further adjustments to the simulation design.

Two samples were fabricated using a vertical centrifugal casting machine. The annealing temperature of one of the heat treatment processes was 817~827 °C, the quenching temperature was 1150~1160 °C, the tempering holding time was 2 h, and the tempering temperature was 550~610 °C. Here, the heat treatment parameters of the actual production quenching temperature of 1180 °C were also referred to, to treat the samples. The microstructure of the samples was then observed using a Quanta 250FEG scanning electron microscope (SEM).

**Figure 10:**
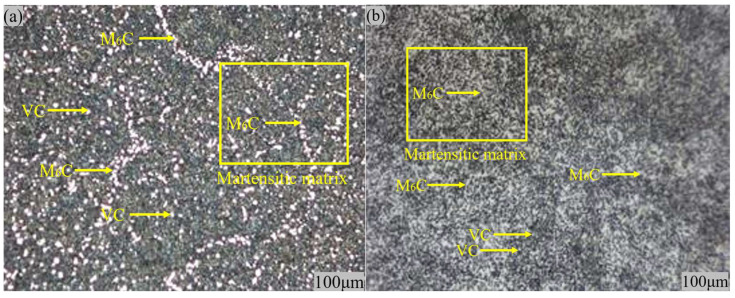
SEM photos after quenching at different temperatures. (**a**) 1180 °C × 100; (**b**) 1160 °C × 100.

**Figure 11:**
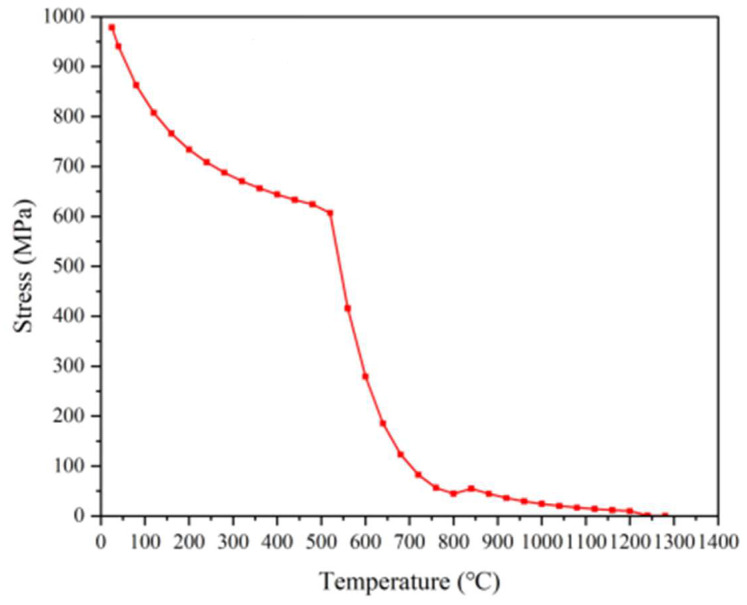
Strength of W-Mo-V high-speed steels at different temperatures.

Figure 10a is the 100-times magnification diagrams of the steel after the HSS material has undergone 1180 °C quenching + two tempering at 560 °C + one tempering at 570 °C. From the diagrams, it is seen that there are a lot of reticulated M_6_C-type carbides and fine granular vanadium carbide distribution uniformly distributed on top of the tempered-martensite matrix, and, due to the further refinement of the austenite in the quenching process grain, it can be found that the martensite matrix grain is also relatively small and uniform. At the same time, after three temperings, the residual austenite is also a great part of the transformation into excellent performance of the tempered martensite. Figure 10b shows 100-times magnification of the steel after quenching at 1160 °C + tempering at 560 °C twice + tempering at 570 °C once. Compared with Figure 10a, the precipitated carbides in the high-speed steel are higher in content, and the M_6_C carbides are also presented in a network as uniformly distributed in the martensitic matrix; the network structure of its M_6_C carbides is more dense and fine, and the vanadium carbide grains are also more fine. The designed HSS performs better in terms of microstructure. From this, it can be preliminarily determined that the performance of the W-Mo-V high-speed steel we designed has been improved. At the same time, as shown in Figure 11, analyzing the high-temperature strength of the material, it can be found that this W-Mo-V-system HSS material has excellent high-temperature strength when the temperature is controlled under 550 °C.

Therefore, this software simulation design test out from the data has an important practical reference significance.

## 6. Conclusions

In this paper, JMatPro software is used to simulate and calculate the equilibrium-phase diagram of W-Mo-V high-speed steel through the analysis of the phase diagram. Additionally, it is used to calculate the W-Mo-V high-speed steel-roll-process parameters and heat-treatment-process parameters, and then the designed parameters and the existing actual production parameters for comparison, to draw the following conclusions.

(1) W-Mo-V high-speed steel belongs to high-alloy steel, in which a large number of alloying elements lead to the existence of a large number and variety of carbides in high-speed steel. These carbides also affect the different properties of high-speed steel, so these alloying elements need to be designed following a certain ratio, to obtain the comprehensive performance of good high-speed steel.
(2) With the increase of carbon content in high-speed steel, the carbon content in the austenite will show a rising trend, and the carbon content in the austenite will not only affect the martensitic transformation temperature, but will also affect the content of residual austenite, so it is necessary to combine many factors to determine the final carbon content.
(3) The use of JMatPro software and empirical formulas to calculate the carbon content of the difference, and even in the actual production, will need to further adjust the carbon content, which is largely due to the actual production process. The air humidity, oxygen content, and temperature will have a certain impact on the performance of high-speed steel.
(4) With the simulation of the W-Mo-V high-speed steel casting temperature of 1610–1660 °C, an annealing temperature of 817–827 °C, a quenching temperature of between 1150 and 1160 °C and a tempering temperature of between 550 and 610 °C, after the appropriate heat treatment process, we can obtain a more refined and uniform or toughened grain. That is, to a certain extent, we can improve the wear resistance, strength, hardness, and toughness of high-speed steel rolls and other comprehensive mechanical properties, to improve the service life of high-speed steel rolls.
(5) The actual production process and the high-speed steel rolls were compared, and we found that the actual production parameters and the design parameters of this paper are not much different, so this paper, through the simulation software calculations of the data obtained by the actual production of a guiding significance, can be used as a theoretical basis in the future research and development of new materials.

## Figures and Tables

**Table 1:** Range of alloy-element content in high-speed steel (wt.%).

Element	C	W	Mo	V	Cr	Si	Mn	Nb	B	N	Fe
Content	1.5–2.5	3.0–7.0	3.0–6.0	4.0–7.5	3.0–6.0	0.5–1.5	0–1.0	0–2.0	0–1.0	0–0.1	Bal.

**Table 2:** Basic chemical composition of commercial HSS powders (wt.%).

Element	C	Cr	Si	V	Mo	W	Mn	Fe
Content	0.94	4.06	0.3	1.9	5.0	5.8	0.3	Bal.

**Table 3:** Effect of carbon content on austenite in high-speed steel.

Carbon Content in High-Speed Steel (%)	Carbon Content in Austenite (%)	Austenitic Peak (%)	Peak Austenite Temperature Range (°C)
1.5	0.35–0.50	86.80	1140–1240
1.6	0.39–0.54	86.30	1130–1230
1.7	0.45–0.59	85.83	1130–1230
1.8	0.50–0.65	85.40	1120–1220
1.9	0.56–0.70	84.98	1120–1220
2.0	0.62–0.76	84.62	1110–1210
2.1	0.68–0.82	84.17	1100–1200
2.2	0.75–0.89	83.97	1090–1190
2.3	0.81–0.95	83.69	1080–1180
2.4	0.89–1.20	83.16	1080–1180
2.5	0.96–1.09	83.23	1070–1170

**Table 4:** Effect of austenite content on martensitic transformation temperature at different temperatures.

Temperature (°C)	Austenite Content (%)	M_s_ (°C)	M50 (°C)	M90 (°C)
1120	82.23	181.3	140.6	46.2
1130	82.50	174.5	133.6	38.5
1140	82.78	167.7	126.4	30.6
1150	83.07	160.7	119.1	22.5
1160	83.37	159.5	117.9	21.2
1170	83.68	177.0	136.2	41.3
1180	83.99	178.1	137.3	42.6
1190	84.31	175.1	134.2	39.2
1200	84.65	172.9	131.9	36.6
1210	84.98	171.8	130.7	35.3
1220	74.16	171.9	130.9	35.5

**Table 5:** W-Mo-V high-speed steel composition content of each alloying element (wt.%).

Element	C	W	Mo	V	Cr	Si	Mn	Nb	B	N	Fe
Content	1.9	5.5	5.0	5.5	4.5	0.7	0.55	0.5	0.2	0.06	Bal

## Data Availability

The original contributions presented in the study are included in the article, and further inquiries can be directed to the corresponding author.
